# Chemical and Antioxidant Properties of Solvent and Enzyme-Assisted Extracts of *Fucus vesiculosus* and *Porphyra dioica*

**DOI:** 10.3390/md22070319

**Published:** 2024-07-18

**Authors:** Paulo Nova, Sara A. Cunha, Ana R. Costa-Pinto, Ana Maria Gomes

**Affiliations:** 1 CBQF—Centro de Biotecnologia e Química Fina Laboratório Associado, Escola Superior de Biotecnologia, Universidade Católica Portuguesa, Rua Diogo Botelho 1327, 4169-005 Porto, Portugal; pnova@ucp.pt (P.N.); scunha@ucp.pt (S.A.C.); amgomes@ucp.pt (A.M.G.); 2 i3S—Instituto de Investigacão e Inovacão em Saúde, Universidade do Porto, 4200-135 Porto, Portugal; 3 IPATIMUP—Instituto de Patologia Molecular e Imunologia da Universidade do Porto, 4200-135 Porto, Portugal

**Keywords:** macroalgae, extraction, carbohydrases, proteases, enzyme-assisted extraction, value-added compounds

## Abstract

Extraction strategies impact the efficiency and nature of extracted compounds. This work assessed the chemical composition and antioxidant capacity of ethanolic, hydroethanolic, and aqueous versus enzyme-assisted extracts (isolated or with the sequential use of alcalase^®^, cellulase^®^, and viscozyme^®^) of the macroalgae *Fucus vesiculosus* (brown, *Phaeophyceae*) and *Porphyra dioica* (red, *Rhodophyta*. For both macroalgae, enzyme-assisted extraction (EAE) was the most efficient process compared to solvent-assisted extraction (SAE), independent of solvent. *Fucus vesiculosus* extraction yields were higher for EAE than for SAE (27.4% to 32.2% and 8.2% to 30.0%, respectively). Total phenolics content (TPC) was at least 10-fold higher in EAE extracts (229.2 to 311.3 GAE/g_extract_) than in SAE (4.34 to 19.6 GAE/g_extract_) counterparts and correlated well with antioxidant capacity (ABTS and ORAC methods), with EAE achieving values up to 8- and 2.6-fold higher than those achieved by SAE, respectively. *Porphyra dioica* followed *F. vesiculosus’s* trend for extraction yields (37.5% to 51.6% for EAE and 5.7% to 35.1% for SAE), TPC, although of a lower magnitude, (0.77 to 8.95 GAE/g_extract_ for SE and 9.37 to 14.73 GAE/g_extract_ for EAE), and antioxidant capacity. Aqueous extracts registered the highest DPPH values for both macroalgae, with 2.3 µmol TE/g_extract_ and 13.3 µmol TE/g_extract_ for *F. vesiculosus* and *P. dioica*, respectively. EAE was a more efficient process in the extraction of soluble protein and reducing sugars in comparison to SAE. Furthermore, an improved effect of enzyme-assisted combinations was observed for almost all analyzed parameters. This study shows the promising application of enzyme-assisted extraction for the extraction of valuable compounds from *F. vesiculosus* and *P.dioica*, making them excellent functional ingredients for a wide range of health and food industrial applications.

## 1. Introduction

The marine environment is full of organisms, extremely rich in bioactive compounds, and has tremendous potential for pharmaceutical, industrial, and biotechnological applications [1,2,3]. Seaweeds stand out as a nutritionally rich sources of compounds such as proteins, polyunsaturated fatty acids, polysaccharides, minerals, pigments, phenolics, and other secondary metabolites with antioxidant, immunomodulatory, anti-cancer, and anti-inflammatory properties [4,5,6,7].

The chemical composition and bioactive properties of seaweeds derive from biotic, environmental, and stress factors such as their species, growth process (cultivation versus wild collection), seasonality (temperature and light intensity), pollutants, geographical distribution, lifecycle, and ecological conditions (available nutrients and salinity) [8,9,10]. Seaweed’s chemical and biological characterization are critical to biotechnological, nutritional, pharmaceutical, and industrial applications. Among the many seaweed species, *Fucus vesiculosus* (brown, *Phaeophyceae*) and *Porphyra dioica* (red, *Rhodophyta*) are highlighted for their important nutritional profiles, in particular, their richness in polysaccharides and phenolics, proteins, dietary fibers, monounsaturated and polyunsaturated fatty acids, as well as minerals such as Ca, Mg, K, Mn, and Fe [11]. Extraction methods play a major role in the type of value-added compounds obtained [6,9,12]. Conventional solid–liquid extraction is the most common yet requires numerous hours to achieve an optimum yield of extracted value-added compounds. Furthermore, when organic solvents are used, the production of environmentally hazardous wastes may occur. For food applications, ethanol, water, or mixtures thereof are the most employed given their low cost and availability, yet extraction efficiency is limited as well as specificity [9,13,14]. Over the past few years, new sustainable green extraction methodologies have been emerging to suppress the carbon footprint, as well as time and resource consuming aspects of conventional extraction. In this context, enzyme-assisted, ultrasound-assisted, pressurized solvent extractions and electro-technologies are being exploited to obtain value-added compounds from macroalgae [9,15,16]. Among these, enzyme-assisted extraction (EAE) offers several advantages, since it does not require organic solvents or harmful chemicals (it is eco-friendly), enables high extraction efficiency and the stability of the extracted compounds, offers reduced extraction times and solvent volumes, allows the conversion of water-insoluble materials to water-soluble materials, and is scalable [9,13,14]. This technology uses hydrolases such as carbohydrases and proteases, which break down a seaweed’s complex cell wall allowing the release of value-added compounds. Parameters such as the pH, temperature, ratio of substrates (solvent: enzyme), and mixing conditions should be considered to improve extraction efficiency [9,14].

**Figure 1:**
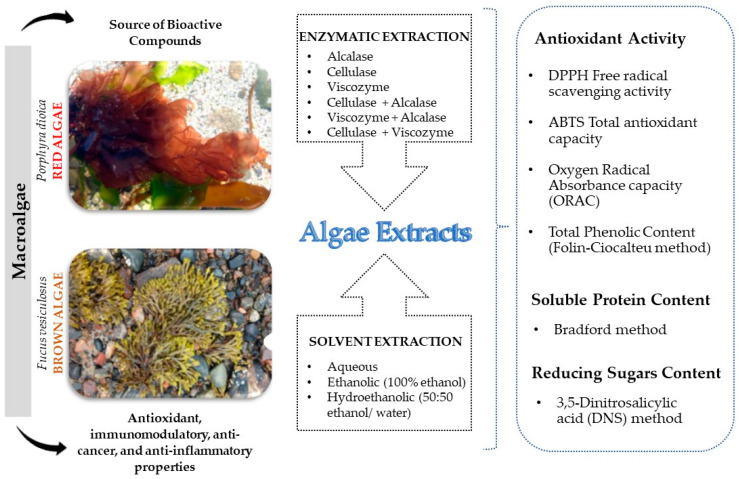
Graphical introduction and methodology.

To date, there is limited research on the application of EAE in seaweeds, specifically *P. dioica* and *F. vesiculosus*, and existing studies have primarily focused on single enzyme applications. This hypothesis-driven study aimed to enhance extraction efficiency by exploring two main objectives: the comparison between solvent-assisted and enzyme-assisted extraction and the evaluation of using a combination of enzymes rather than single enzyme applications (Figure 1). To evaluate the extraction efficiency of value-added compounds from these seaweeds by EAE against conventional aqueous/ethanol-assisted extraction, aqueous, ethanolic (100% ethanol), hydroethanolic (50:50, ethanol/water), and enzyme-assisted extractions were conducted on *F. vesiculosus* and *P. dioica*. The resulting extracts were analyzed for their extraction yield, antioxidant capacity, phenolic, soluble protein, and reducing sugars content. In the case of EAE, to assess the improved effect of enzyme sequential use, a protease—alcalase^®^, and two cell wall degrading enzymes—cellulase^®^ (carbohydrase) and viscozyme^®^ (a multi-enzyme complex containing a wide range of carbohydrases including arabanase, cellulase, β-glucanase, hemicellulase, and xylanase)—were tested either individually or in combination.

## 2. Results and Discussion

### 2.1. Extraction Yield (%)

The extraction efficiency of value-added compounds from macroalgae requires the application of specific enzymes, such as proteases and carbohydrases with the capacity to degrade the chemically complex and heterogeneous algal cell wall leading to the release of valuable compounds from seaweeds [14]. Solvents of different polarity can also be used effectively to extract compatible soluble compounds [17]. Several solid–liquid extraction techniques can be used to extract intracellular natural products from microorganisms, plants, or macroalgae [17]. Solid–liquid extraction has the advantage of the higher solubilities of compounds in different solvents; however, these methods are typically time-consuming and require large amounts of non-biodegradable and toxic organic solvents [17,18].

**Figure 2:**
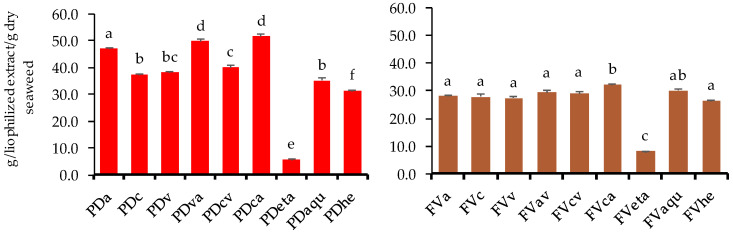
Extraction yields of *P. dioica* (PD) and *F. vesiculosus* (FV) extracts obtained by enzyme-assisted extraction with alcalase (Pda; FVa), cellulase (PDc; FVc), viscozyme (PDv; FVv), viscozyme + alcalase (PDva; FVva), cellulase + viscozyme (PDcv; FVcv), cellulase + alcalase (PDca; FVca) and ethanolic (PDeta; FVeta), aqueous (PDaqu; FVaqu), and hydroethanolic (PDhe; FVhe) extractions. Different letters indicate significant differences (*p* < 0.05) between extracts for each seaweed (n = 3).

The effect of the different extraction methods on the yield is presented in Figure 2 for both *P. dioica* and *F. vesiculosus*. In general, higher extraction yields were observed when extraction was assisted by the coupling of both types of hydrolytic enzymes (carbohydrases and proteases). Furthermore, an effect of seaweed species on the extraction yield was also observed. For *F. vesiculosus*, the highest extraction yields ranged between 27.38 and 32.21% and were achieved for enzyme-assisted and aqueous extracts (30.00%). Enzyme combinations were revealed to be more effective in comparison with isolated enzyme-assisted extraction, with higher values obtained for the cellulase and alcalase mixture (FVca) (32.21%). Furthermore, the FVca extraction efficiency was statistically different in comparison with all other extraction methods assessed except for comparisons with aqueous extraction. These results are higher than those of the extraction yield presented by Getachew et al. (2022), for the low polarity water extraction [a green extraction technology that relies on keeping the critical temperature (374 °C) and boiling point (100 °C) at a very high pressure to keep water in its liquid form] of wild *F. vesiculosus* (harvested in Denmark) (25.99%) [19]. Because of these conditions, the water acquires a low viscosity, a high dissociation constant (kw), a low dielectric constant, a low surface tension, and could be compared with commonly used organic solvents such as ethanol and methanol [19,20]. On the other hand, in the case of the red macroalga *P. dioica*, the highest extraction yields were almost two-fold higher than those of the brown *F. vesiculosus* (Figure 2). The highest extraction yields were achieved for the enzyme-assisted extraction with alcalase (PDa), the viscozyme + alcalase mixture (PDva), and the cellulase + alcalase mixture (PDca), with 46.97%, 49.76%, and 51.62%, respectively. These results indicate that alcalase is the main component responsible for the extraction efficiency since the use of this proteolytic enzyme led to higher extraction yields in comparison with cellulase and viscozyme used alone or in combination. Furthermore, the use of alcalase mixed with cellulase or viscozyme leads to an improved effect with higher extraction yields (Figure 2). The simultaneous or sequential combination of alcalase with other proteases has been described as an effective methodology to extract large amounts of peptides, but its combination with cellulolytic activity enzymes is an innovative approach to extract different compounds with biological activity from macroalgae [21]. Regarding solvent extraction, aqueous extracts presented higher extraction yields for both macroalgae (30% and 35.1%, for *F. vesiculosus* (FVaqu) and *P. dioica* (PDaqu), respectively), in comparison with ethanolic (8.2% and 5.7% for FVaqu and PDaqu, respectively) and hydroethanolic extractions (26.5% and 31.5% for FVaqu and PDaqu, respectively) (Figure 2). Similar results regarding higher extraction yields from aqueous extraction in comparison with hydroethanolic extractions were presented by Félix et al. (2020) for the extraction of *Grateloupia turuturu,* an invasive red macroalga on the Iberian coast, first reported in Portugal in 1997 [22,23]. Regarding *G. turuturu*, the authors expected these results since water was capable of extracting sulfated galactans as well as the protein fraction from the algae biomass [22]. A similar trend could be happening with the results from the present study since *P. dioica* presents considerable amounts of sulfated galactans, and both *F. vesiculosus* and *P. dioica* present considerable amounts of protein [11].

### 2.2. Total Antioxidant Capacity

Antioxidant compounds play an important role in antagonizing various diseases and aging biochemical processes and, as such, their potential for food, medicine, and cosmetic applications is increasingly growing [3,24]. The total phenolic content of marine macroalgae has been correlated with their antioxidant activity [25,26]. This parameter is influenced by several factors such as species, age, growth stage, geographical location, nutrient availability, and sunlight exposure, among others; therefore, an extensive knowledge of the environmental impact is crucial to fully understand macroalgae’s biological and biochemical variability [27].

**Figure 3:**
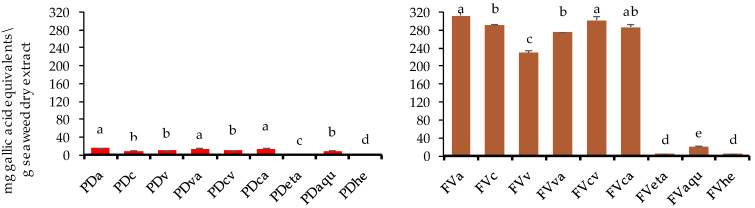
Total phenolic compound contents (Folin–Ciocalteu) of *P. dioica* (PD) and *F. vesiculosus* (FV) extracts obtained by enzyme-assisted extraction with alcalase (Pda; FVa), cellulase (PDc; FVc), viscozyme (PDv; FVv), viscozyme + alcalase (PDva; FVva), cellulase + viscozyme (PDcv; FVcv), cellulase + alcalase (PDca; FVca), and ethanolic (PDeta; FVeta), aqueous (PDaqu; FVaqu), and hydroethanolic (PDhe; FVhe) extractions. Different letters indicate significant differences (*p* < 0.05) between extracts for each seaweed (n = 3).

In the present study, total phenolic content varied significantly according to the extraction method used, with higher values obtained for the enzyme-assisted extracts (*p* < 0.05). Enzyme-assisted extraction proved to be more efficient than ethanolic, hydroethanolic, and aqueous extraction processes (Figure 3). Regarding the macroalgae *F. vesiculosus*, the highest contents of total phenolic compounds were achieved for the enzyme-assisted extraction with alcalase (FVa), cellulase (FVc), and cellulase + viscozyme mixture (FVcv) (311.30, 291.97, and 301.03 mg GAE/g dry seaweed extract, respectively). Habeebullah et al. (2021) investigated the effect of enzyme-assisted extraction on four brown wild Danish seaweeds, *F. vesiculosus*, *F. serratus*, *Ascophyllum nosodum*, and *Polysiphonia fucoides*, applying five carbohydrases (viscozyme, Celluclast, AMG, Termamyl, and Ultraflo) and three proteases (Neutrase, Flavourzyme, and alcalase) [28]. The authors obtained lower total phenolic content values when compared with the ones of the present study for *F. vesiculosus* extraction with alcalase (10.7 mg GAE/g extract) and viscozyme (29.2 mg GAE/g dry extract) [28]. Furthermore, the *F. vesiculosus* total phenolic content is higher in comparison with the other algae from the brown genus *F. serratus* (29.3 to 30.7 mg GAE/g extract), *Ascophyllum nosodum* (10.1 to 22.1 mg GAE/g dry extract) and *Polysiphonia fucoides* (10.4 to 18.5 mg GAE/g dry extract) [28]. Despite the high values reported herein, a future optimization process of the enzyme-assisted extracts may need to focus on the temperature as a variable to further increase the total phenolic content extraction of *F. vesiculosus*. Moreover, the temperature will need to be carefully controlled since high temperatures are correlated with a reduction in the total phenolic content of seaweeds [29]. Considering the comparison between enzyme-assisted extractions with aqueous/ethanolic extractions, indeed, total phenolic contents in the enzyme-assisted extracts were ca. fifteen-fold higher than those in the aqueous extracts (19.6 mg GAE/g dry seaweed extract). Nonetheless, the results achieved for aqueous extracts were similar or even higher than those reported in the literature. For example, Ref. [19] performed a low-polarity water extraction of wild *F. vesiculosus* at different temperatures between 120 and 200 °C and reported total phenolic content values that ranged from approximately 1.8 to 12.2 mg GAE/g dry seaweed extract, respectively [19]. On the other hand, Nunes et al. (2022) prepared two different aqueous extractions (decoction at 100 °C for 30 min and 24 h extraction at 25 °C) of different gender and growth stages (<20 to >30 cm) of *F. vesiculosus* collected in Tagus Estuary Natural Reserve, Lisbon [30], and achieved higher results in decoction at 100 °C for 30 min—which slightly surpassed the 40 µg phloroglucinol equivalents/mg of seaweed for the female gender and 20–25 cm, 25–30 cm, and >30 cm sized *F. vesiculosus* [30].

Total phenolic content also varied significantly among species types and results obtained for the red macroalgae *P. dioica* did not go above 15 mg GAE/g dry seaweed extract. The highest total phenolic content extraction was achieved for the enzyme-assisted procedures with alcalase (PDa), the viscozyme + alcalase mixture (PDva), and the celulase + alcalase mixture (PDca), with similar (*p* > 0.05) values of 14.73, 14.09, and 14.15 mg GAE/g dry seaweed extract, respectively (Figure 3). Regarding extraction efficiency, alcalase alone or in combination proved to be the most efficient in extracting phenolic compounds from the macroalgae *P. dioica*. Contrary to what was previously observed for the combination of enzymes, in this case, the combination of protease and cellulase did not lead to an improved effect, with the highest efficiency being achieved with alcalase extraction alone (Figure 3). The results achieved in the present study are higher than the ones presented by Carpena et al. (2021) for the heat-assisted extraction of the red macroalgae *Chondrus crispus*, *Mastocarpus stellatus*, and *Gigartina pistillata* collected from the natural environment along the Pontevedra coasts (Galicia, Spain) and for which total phenolic contents were 10.36, 12.18, and 9.11 mg/g dry weight [31].

**Figure 4:**
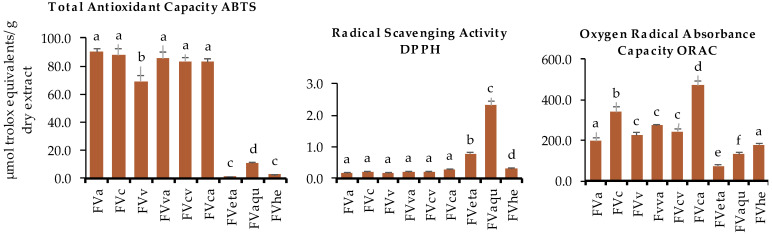
Total antioxidant capacity (ABTS), DPPH free radicals and the oxygen radical absorbance capacity (ORAC) of *F. vesiculosus* (FV) extracts obtained by enzyme-assisted extraction with alcalase (FVa), cellulase (FVc), viscozyme (FVv), viscozyme + alcalase (FVva), cellulase + viscozyme (FVcv), cellulase + alcalase (FVca), and ethanolic (FVeta), aqueous (FVaqu), and hydroethanolic (FVhe) extraction. Different letters indicate significant differences (*p* < 0.05) between extracts (n = 3).

**Figure 5:**
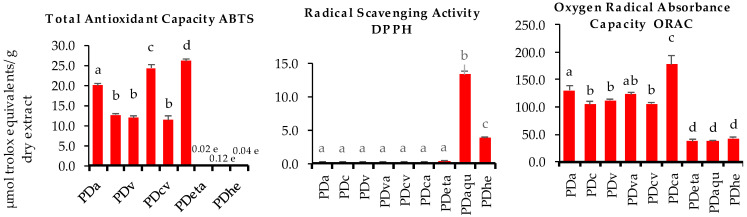
Total antioxidant capacity (ABTS), DPPH free radicals and the oxygen radical absorbance capacity (ORAC) of *P. dioica* (PD) extracts obtained by enzyme-assisted extraction with alcalase (FVa), cellulase (FVc), viscozyme (FVv), viscozyme + alcalase (FVva), cellulase + viscozyme (FVcv), cellulase + alcalase (FVca), and ethanolic (FVeta), aqueous (FVaqu), and hydroethanolic (FVhe) extraction. Different letters indicate significant differences (*p* < 0.05) between extracts (n = 3).

The antioxidant activity of the seaweed extracts was measured by three different in vitro assays (ABTS total antioxidant capacity, DPPH free radical, and oxygen radical absorbance capacity (ORAC)). Results showed that antioxidant activity changed with the application of the different enzymes, assessed alone or in combination, for both algae (Figure 4 and Figure 5). For total antioxidant capacity, *F. vesiculosus* and *P. dioica* presented higher results regarding the enzyme-assisted extractions in comparison with ethanolic, hydroethanolic, or aqueous procedures, although a larger variability was observed among these extractions in the case of *P. dioica*. For *F. vesiculosus*, total antioxidant capacity values ranged from 69.1 to 90.3 µmol TE/g dry seaweed extract for viscozyme (FVv) and alcalase extraction (FVa), respectively. In addition, no statistically significant differences were observed for comparisons between celulase (FVc), alcalase (FVa), and extractions with enzyme-assisted combinations (FVva, FVcv, and FVca). Concerning the total antioxidant capacity of *P. dioica* enzyme-assisted extractions, absolute values were at least three times lower than those observed for *F. vesiculosus* within the same extraction procedure and varied from 11.4 to 26.1 µmol TE/g dry seaweed extract for cellulase + viscozyme (PDcv) and cellulase + alcalase (PDca) combinations, respectively. When comparing each of the three enzymes used in single form, alcalase extraction (PDa) was revealed to be the most efficient, and an improved effect from the enzyme-assisted combination of alcalase both with cellulase (PDca) or viscozyme (PDva) was observed (Figure 5). Concerning DPPH radical scavenging activity and ORAC values, both macroalgae revealed similar trends. Aqueous extraction was more efficient for the extraction of compounds with the capacity to scavenge DPPH free radicals (13.3 and 2.3 µmol TE/g dry seaweed extract for *P. dioica* and *F. vesiculosus*, respectively) (Figure 4 and Figure 5). Rodrigues, Sousa et al. (2015) also reported variable DPPH scavenging activities in brown and red seaweed extracts obtained by carbohydrases or proteases [32]. ORAC presented interesting results and the highest extraction efficiency of both macroalgae regarding this parameter was achieved for the cellulase + alcalase combination (PDca—178.2 µmol TE/g dry seaweed extract; FVca—471.2 µmol TE/g dry seaweed extract) (Figure 4 and Figure 5). These values are higher in comparison with ORAC values presented for tropical fruits such as pineapple, sugar apple, papaya fruit, longan, mangosteen, lychee, langsat, mango, rambutan, and guava, which were 33.55, 30.88, 38.10, 51.01, 38.62, 21.44, 23.13, 16.27, 22.57, 21.83, 15.58, and 69.62 µmol TE/g dry weight, respectively [33]. This data highlights the potential of these marine resources to be incorporated daily into a healthy diet and as novel ingredients for the development of innovative functional foods.

As expected, a positive correlation between total phenolic content values and antioxidant activity was observed. For both macroalgae, extraction methods with higher total phenolic contents presented the highest antioxidant activity in almost all assays. This indicates that these extraction methods were capable of extracting phenolic compounds with the capacity for scavenging free radicals. The same conclusions regarding the correlation between the higher total phenolic content and the antioxidant activity of macroalgae extracts were previously described by other authors [25,34,35]. Overall, these macroalgae in their composition and functionality may constitute alternative and sustainable ingredients of marine origins to be incorporated in the formulation of innovative food products that privilege health and nutrition, also targeting environmental sustainability.

### 2.3. Soluble Protein

Algae are considered a valuable source of protein, covering all the essential amino acids, yet contents vary according to previously described biotic, environmental, and stress factors, as well as extraction methods [8,9]. The soluble protein contents of macroalgae enzyme-assisted and solvent extracts are presented in Table 1. For both macroalgae, enzyme-assisted extraction proved to be a more efficient method for the extraction of soluble protein in comparison with solvent extractions with water, ethanol, and water/ethanol in combination. For *F. vesiculosus*, alcalase extraction for 24 h (FVa) presented a higher soluble protein content (6.31 BSA eq/100 g dry seaweed extract) in comparison with the other enzyme-assisted extraction procedures. This protein content is higher than the one presented by Soares et al. (2021) for the multi-step subcritical water extraction of *F. vesiculosus*, also produced in an integrated multi-trophic aquaculture system and supplied by ALGAplus and for which the content was 3.1 g/100 g dry weight [36]. A similar trend can be observed in comparison with the distilled water extract of *Codium tomentosum*, another alga from the brown genus, also produced by ALGAplus, whose content was 0.09 g/100 g (% *w*/*w* dry seaweed) [37]. In our study, an alcalase sequential combination of *F. vesiculosus* with either cellulase (FVca) or viscozyme (FVva) for 12 + 12 h showed an improved effect in comparison with the standalone use of cellulase (FVc) or viscozyme (FVv) (5.44 and 5.28 BSA eq/100 g dry seaweed extract vs. 3.87 and 3.69 BSA eq/100 g dry seaweed extract, respectively). Based on these results, future studies should explore this improved effect considering the ratio of substrates (solvent: enzymes) and extraction time for each enzyme.

For the red macroalgae *P. dioica*, sequential enzyme-assisted extraction showed a higher efficiency for soluble protein extraction in comparison with the standalone use of enzymes with the higher contents observed for extraction with viscozyme + alcalase (PDva), cellulase + viscozyme (PDcv), and cellulase + alcalase (PDca) extractions with 3.80, 3.94, and 3.42 BSA eq/100 g dry seaweed extract, respectively. These results are higher than the ones obtained by Ferreira et al. (2022) for *P. dioica* also produced in an integrated multi-trophic aquaculture system and supplied by ALGAplus extracted with distilled water and for which the protein content was 0.41 g/100 g (% *w*/*w* dry seaweed) [37]. The same conclusions were observed in *Gracilaria gracilis*, also from the red genus and studied by Ferreira et al. (2022) whose soluble protein content was 0.15 g/100 g (% *w*/*w* dry seaweed) [37].

In comparison with *F. vesiculosus* extraction, cellulase used alone was not as efficient concerning the extraction of soluble protein from the macroalgae *P. dioica.* These results are probably due to the different cell wall compositions of both seaweeds: cellulose, pectins and polysulphate esters in the case of *P. dioica* and fucoidan, alginate, and cellulose for *F. vesiculosus* [4,11]. Macroalgae have a rigid cell wall and to overcome this problem, it is necessary to develop strategies to weaken the wall, allowing proteases to access intracellular protein for hydrolysis. It was noted that the application of carbohydrases (cellulase or viscozyme) on both macroalgae allowed a robust breaking of the cell wall and a more efficient release of proteins with further alcalase application.

### 2.4. Reducing Sugars Content

For both macroalgae, enzyme-assisted extraction proved to be an efficient method for the extraction of reducing sugars in comparison with solvent extractions with water, ethanol, and water/ethanol in combination. Results are presented in Table 1. For the macroalgae *F. vesiculosus*, higher reduced sugar contents were achieved for extractions with cellulase (FVc) and the cellulase + viscozyme combination (FVcv) with 12.2 and 12.1 g/100 g dry seaweed extract, respectively; these similar values for both simple or combined extraction procedures reflect the higher impact of cellulase. These results are higher than the ones observed by Soares et al. (2021) for the subcritical water extraction of *F. vesiculosus* fractions for which the reducing sugars content ranged from 0.3 to 1.1 g/100 dry weight [36]. Similar conclusions can be taken in comparison with distilled water extract from the brown macroalgae *Alaria esculenta* (2.43 g/100 g (% *w*/*w* dry seaweed)) and subcritical water extracts from *Codium tomentosum* (results ranged from 0.1 to 1.8 g/100 g (% *w*/*w* dry seaweed)) [36,37].

For the extraction of *P. dioica*, procedures with cellulase (PDc), viscozyme (PDv), and their combination (PDcv) achieved higher results with 7.6, 7.4, and 8.3 g/100 g dry weight, respectively. In this case, an improved effect of both carbohydrases was observed (Table 2). Our results are higher in comparison with *P. dioica* distilled water extract performed by Ferreira et al., 2022, for which the content was 0.24 g/100 g (% *w*/*w* dry seaweed), as well as with comparisons with *Gracilaria* sp. (an alga from the red genus) for which reducing sugars content were 0.19 g/100 g (% *w*/*w* dry seaweed) [37]. Once again, the more effective rupture of the cell wall with the use of carbohydrases may support these higher values.

## 3. Materials and Methods

### 3.1. Chemicals and Reagents

The enzymes cellulase from *Trichoderma* sp. (enzymatic activity: ≥5000 units/g solid), viscozyme (cell wall-degrading enzyme complex from *Aspergillus* sp., lysing enzyme from *Aspergillus* sp.; enzymatic activity: ≥100 FBGU/g), and alcalase (*Bacillus licheniformis;* enzymatic activity: ≥0.75 Anson units/mL) were obtained from Sigma-Aldrich (St. Louis, MO, USA). Gallic acid was obtained from LabChem (Zelienople, PA, USA). Folin & Ciocalteu′s phenol reagent, fluorescein, 2,2′-azo-bis-(2-methylpropionamidine)-dihydrochloride (≥97%), 6-hydroxy-2,5,7,8-tetramethylbroman-2-carboxylic acid (Trolox) (≥97%), 2,2′-Azino-bis(3-ethylbenzothiazoline-6-sulfonic acid) diammonium salt (ABTS), 2,2-Diphenyl-1-picrylhydrazyl (DPPH), absolute ethanol (CH3CH2OH), and 3,5-Dinitrosalicylic acid (DNS) were obtained from Sigma-Aldrich (St. Louis, MO, USA). The Coomassie (Bradford, UK) Protein Assay Kit was obtained from Thermo Fisher Scientific (Waltham, MA, USA).

### 3.2. Algal Supply

*Porphyra dioica* and *Fucus vesiculosus* were obtained from land-based, fully controlled cultivation systems under the integrated multi-trophic aquaculture sustainable concept and provided by ALGAplus^®^ (a specialized Portuguese company in the production and commercialization of seaweeds for food and cosmetic applications located at the coastal lagoon of Ria de Aveiro). The same batch of each seaweed was used throughout the different experiments to minimize biomass variability. Both seaweed batches were kindly provided in dried homogeneous powder with a less than 1.0 mm particle size.

### 3.3. Aqueous and Ethanolic Extract Preparation

Aqueous and ethanolic extracts of each alga were prepared according to the method described by Haq et al. [38] with some modifications. Dried powder *P. dioica* and *F. vesiculosus* algae samples (2 g) were soaked in either 50 mL of absolute ethanol, ultrapure water, or 50:50 ethanol/purified water at 25 °C for 24 h under slow, constant agitation (100 rpm). The mixtures were then sonicated 5 times for 30 s at 130 W, 20 kHz using the ultrasonic processor sonics VCX 130 and centrifuged at 5000× *g* for 10 min at 4 °C. Solvents were evaporated using a rotavapor (Buchi R-210 Rotavapor System). The resulting extracts were freeze-dried and stored at −20 °C for further experiments. All extraction procedures were conducted in triplicate. A stock solution of 10 mg/mL of each algae extract was used for the subsequent studies.

### 3.4. Enzyme-Assisted Extraction

The enzyme-assisted extracts from *F. vesiculosus* and *P. dioica* were performed with two carbohydrate-degrading enzymes (viscozyme and cellulase) and one protease (alcalase). The enzyme-assisted hydrolyses were performed according to the methods described by Rodrigues et al., 2015, with some modifications [32]. The selected enzymes were assessed isolated or in combination as described in Table 3, where the different experimental conditions applied are shown. For the enzyme-assisted extraction, 2 g of dried seaweed was added to 50 mL of ultrapure water. Then, the pH was adjusted to the optimal pH of the selected enzyme using 1 M of NaOH and/or 1 M of HCl (Table 3). The enzyme [50 μL of viscozyme (6 FBGD/g of seaweed) or of alcalase (37.5 U/g of seaweed or 50 µg of cellulase (250 U/g of seaweed)] was added to the mixture and incubated at 50 °C in an orbital for 24 h in the dark at 125 rpm. For cellulase + alcalase and viscozyme + alcalase enzyme combinations, the pH was initially adjusted for cellulase and viscozyme to be the optimal condition (5.0), the respective enzyme was added, and the mixture was incubated for 12 h. Subsequently, the pH was adjusted for alcalase to be an optimal condition (8.0), the alcalase was added, and the resulting mixture was incubated for an additional period of 12 h. All pH conditions were optimized in previous studies. After hydrolysis, the reaction was ended by boiling the samples at 100 °C for 10 min followed by immediate cooling with ice. The hydrolysate was centrifuged at 5000× *g* for 10 min at 4 °C. The supernatant was subsequently transferred to a plastic container and freeze-dried. The freeze-dried powder was stored at −20 °C for further experiments. All extraction procedures were conducted in triplicate.

### 3.5. Extraction Yield

The total extraction yield for each seaweed extract was determined according to the following equation:

Extraction yield (%) = m1/m0 × 100

where m1 is the total mass of the freeze-dried seaweed extract, and m0 is the initial mass of the dried macroalgae used in each extraction.

All subsequent analyses were performed using three true replicates and analyzed in triplicate (n = 9).

### 3.6. Extract Characterization

#### 3.6.1. Total Phenolic Content

The total phenolic content (TPC) was determined by the method of Folin–Ciocalteu as previously described by Rodrigues, Sousa et al., 2015 [32], using gallic acid as the standard (0.025–0.200 mg/mL final concentration in well). Results were expressed as micrograms of gallic acid equivalents per gram of dry seaweed extract (GAE/g extract). The TPC was determined by colorimetry at 720 nm (Synergy H1; BioTek Instruments, Winooski, VT, USA).

#### 3.6.2. Total Antioxidant Capacity

##### DPPH Free Radical Scavenging Activity

The DPPH free radical scavenging activity was determined according to the method described by Suresh et al., 2013, using Trolox as the standard (25–250 µM, final concentration in well) [39]. An aliquot (0.1 mL) of each extract (10 mg of lyophilized solids/mL) was added to 3.0 mL of a 0.1 mM ethanolic DPPH solution. After incubation for 30 min at 30 °C in the dark, the absorbance was measured at 517 nm (Synergy H1; BioTek Instruments, Winooski, VT, USA). Results were expressed as the equivalent concentration of Trolox (µmol TE/g dry seaweed).

##### ABTS Free Radical Scavenging Activity

The total antioxidant capacity of the extract solutions was also measured according to the method described by Gião et al., 2007 [40]. A total of 2 mL of diluted ABTS solution was added to 120 μL of the extract, and the absorbance at 734 nm (Synergy H1; BioTek Instruments, Winooski, VT, USA) was measured using Trolox as the standard (25–250 µM, final concentration in well). Results were expressed as µmol TE/g dry seaweed extract.

##### Oxygen Radical Absorbance Capacity

The oxygen radical absorbance capacity (ORAC) assay was performed in a black polystyrene 96-well microplate (Nunc, Denmark) according to the method described by Coscueta et al., 2020 [41]. The experiment was carried out in 75 mM of a phosphate buffer (pH 7.4). The final reaction mixture was 200 µL— (seaweed extract (20 µL) and fluorescein (120 µL) 70 nM, final concentration in well) solutions were placed in the well of the microplate. The mixture was preincubated for 10 min at 37 °C. An AAPH solution (60 µL; 12 mM, final concentration in well) was added rapidly. The microplate was immediately placed in the reader and the fluorescence was recorded at intervals of 1 min for a total of 80 min. A multidetector plate reader (Synergy H1; BioTek Instruments, Winooski, VT, USA) with excitation and emission wavelengths of 485 nm and 528 nm, respectively, was used to monitor the reaction. Trolox (1–8 µM, final concentration in well) was used as the standard for the calibration curve. A blank (fluorescein + AAPH) using a phosphate buffer instead of the antioxidant solution was performed. The microplate was automatically shaken before each reading. Trolox and AAPH solutions were prepared daily, and fluorescein was diluted from a stock solution (1.17 mM) in the same phosphate buffer. Antioxidant curves (fluorescence versus time) were first normalized to the curve of the blank corresponding to the same assay by multiplying original data by the factor fluorescence_blank,t=0_/fluorescence_control,t=0_. From the normalized curves, the area under the fluorescence decay curve (AUC) was calculated according to the trapezoidal method. The final AUC values were calculated by subtracting the AUC of the blank from all the results. Regression equations between net AUC an d antioxidant concentration were calculated. Results were expressed in µmol TE/g dry seaweed extract.

#### 3.6.3. Soluble Protein—Bradford Method

Soluble protein content was determined by the Bradford assay (1976) with a slight modification, as previously described by Gómez-García et al. (2021) [42,43]. In this method, 0.950 mL of Bradford reagent was combined with 0.05 mL of each seaweed sample at room temperature (20 ± 0.5 °C). The reaction was incubated in the dark for 20 min. Absorbance was measured at 595 nm (Synergy H1; BioTek Instruments, Winooski, VT, USA) and protein concentration was calculated with a calibration curve using bovine serum albumin (BSA) as the standard (25–2000 μg/mL). Results were expressed as g BSA equivalents/100 g dry seaweed extract.

#### 3.6.4. Reducing Sugars Measurements

The 3,5-Dinitrosalicylic acid (DNS) method was performed to estimate the amount of reducing sugars in the seaweed extracts according to the method developed by Miller (1959), adapted to the 96-well microplate scale by Gonçalves et al. (2010) [44,45]. In this method, the yellow 3,5-dinitrosalicylic acid is reduced to orange 3-amino-5-nitrosalicylic acid by the action of the free aldehyde and ketone groups of the seaweed samples’ reducing sugars. The reaction was performed using a 96-well microplate (Nunc, Denmark) and in a multidetector plate reader (Synergy H1; BioTek Instruments, Winooski, VT, USA). In each well, 25 µL of sample and 25 µL of dinitrosalicylic acid (DNS) were added and the plate was incubated at 100 °C for 5 min in a water bath. After cooling on ice, 250 μL of distilled water was added and the absorbance was read at 540 nm. The calibration curve was obtained using standard solutions with glucose concentrations ranging from 0 to 2.0 g/L.

### 3.7. Statistical Analysis

Results are reported as mean values ± standard deviations. Statistical analysis was performed using IBM SPSS Statistics version 29 for Microsoft Windows. Normality and homogeneity were examined and a One-way ANOVA with the Holm–Sidak test for post hoc analyses was applied to evaluate statistical differences between enzyme-assisted, aqueous, hydroethanolic, and ethanolic extractions for each seaweed (*p* < 0.05).

## 4. Conclusions

This study shows the promising application of enzyme-assisted extraction, alone or in specific combinations, for the extraction of valuable compounds from seaweeds; this process was shown to be a more efficient process compared to ethanolic, aqueous, and hydroethanolic extraction methods. Furthermore, an improved effect of enzyme-assisted combinations was observed for almost all analyzed parameters. For both macroalgae, higher antioxidant profiles were obtained with the sequential use of enzymes with cellulolytic and proteolytic activity. Regarding soluble protein extraction, enzyme-assisted combinations achieved higher results for both macroalgae, except for *F. vesiculosus* extraction with alcalase alone. For the extraction of reducing sugars, the sequential or isolated use of carbohydrases proved to be the most efficient procedure. The remarkable potential of these marine macroalgae extracts makes them excellent ingredients for a wide range of health, food, and industrial applications. Both *F. vesiculosus* and *P. dioica* present an important antioxidant profile as well as considerable soluble protein and reducing sugars content which are suitable for the development of novel functional foods.

## Figures and Tables

**Table 1:** Soluble protein contents of *F. vesiculosus* and *P. dioica* ethanolic, hydro-ethanolic, aqueous, and enzyme-assisted extracts.

Enzymatic assisted Extracts	***F. vesiculosus***	**g BSA Equivalents/100 g Dry Seaweed Extract**	***P. dioica***	**g BSA Equivalents/100 g Dry Seaweed Extract**
	FVa	6.31 ± 0.31 ^a^	PDa	2.79 ± 0.03 ^a^
	FVc	3.87 ± 0.10 ^b^	PDc	3.34 ± 0.18 ^b^
	FVv	3.69 ± 0.18 ^b^	PDv	2.39 ± 0.14 ^c^
	FVva	5.28 ± 0.29 ^c^	PDva	3.80 ± 0.05 ^d^
	FVcv	4.79 ± 0.23 ^d^	PDcv	3.94 ± 0.30 ^d^
	FVca	5.44 ± 0.03 ^c^	PDca	3.42 ± 0.12 ^b^
Solvent Extracts	Ethanolic	0.94 ^e^	Ethanolic	0.90 ^e^
	Aqueous	2.90 ^f^	Aqueous	1.60 ^f^
	Hydro-ethanolic	0.79 ^e^	Hydro-ethanolic	0.73 ^e^

Enzyme-assisted extraction with alcalase (Pda; FVa), cellulase (PDc; FVc), viscozyme (PDv; FVv), viscozyme + alcalase (PDva; FVva), cellulase + viscozyme (PDcv; FVcv), cellulase + alcalase (PDca; FVca). ^a–f^ in a column: different letters indicate significant differences (*p* < 0.05) between extraction procedures. BSA: Bovine serum albumin n = 3.

**Table 2:** Reducing sugars content of *F. vesiculosus* and *P. dioica* enzymatic-assisted and solvent extracts.

Enzymatic assisted Extracts	***F. vesiculosus***	**g/100 g Dry Seaweed Extract**	***P. dioica***	**g/100 g Dry Seaweed Extract**
	FVa	7.3 ± 0.3 ^a^	PDa	5.2 ± 0.3 ^a^
	FVc	12.2 ± 0.3 ^b^	PDc	7.6 ± 0.6 ^b^
	FVv	5.3 ± 0.3 ^c^	PDv	7.4 ± 0.2 ^b^
	FVva	6.7 ± 0.1 ^d^	PDva	5.2 ± 0.2 ^a^
	FVcv	12.1 ± 0.4 ^b^	PDcv	8.3 ± 0.3 ^c^
	FVca	8.1 ± 0.3 ^e^	PDca	5.6 ± 0.3 ^a^
Solvent Extracts	Ethanolic	0.75 ± 0.03 ^f^	Ethanolic	0.57 ± 0.03 ^d^
	Aqueous	1.98 ± 0.4 ^g^	Aqueous	1.20 ± 0.2 ^e^
	Hydro-ethanolic	0.87 ± 0.06 ^f^	Hydro-ethanolic	0.65 ± 0.02 ^d^

Enzyme-assisted extraction with alcalase (Pda; FVa), cellulase (PDc; FVc), viscozyme (PDv; FVv), viscozyme + alcalase (PDva; FVva), cellulase + viscozyme (PDcv; FVcv), cellulase + alcalase (PDca; FVca). ^a–g^ in a column: different letters indicate significant differences (*p* < 0.05) between extraction procedures n = 3.

**Table 3:** Enzyme-assisted hydrolysis conditions of macroalgae extracts at 50 °C.

Enzymes	Time (hours)	pH
Alcalase	24	8.0
Cellulase	24	5.0
Viscozyme	24	5.0
Cellulase + viscozyme	24	5.0
Cellulase + alcalase	12 + 12	5.0/8.0
Viscozyme + alcalase	12 + 12	5.0/8.0

## Data Availability

All data generated or analyzed during this study are included in this published article.
